# A Multilevel Gamma-Clustering Layout Algorithm for Visualization of Biological Networks

**DOI:** 10.1155/2013/920325

**Published:** 2013-06-26

**Authors:** Tomas Hruz, Markus Wyss, Christoph Lucas, Oliver Laule, Peter von Rohr, Philip Zimmermann, Stefan Bleuler

**Affiliations:** ^1^Institute of Theoretical Computer Science, ETH Zurich, 8092 Zurich, Switzerland; ^2^NEBION AG, Hohlstraße 515, 8048 Zurich, Switzerland

## Abstract

Visualization of large complex networks has become an indispensable part of systems biology, where organisms need to be considered as one complex system. The visualization of the corresponding network is challenging due to the size and density of edges. In many cases, the use of standard visualization algorithms can lead to high running times and poorly readable visualizations due to many edge crossings. We suggest an approach that analyzes the structure of the graph first and then generates a new graph which contains specific semantic symbols for regular substructures like dense clusters. We propose a multilevel gamma-clustering layout visualization algorithm (MLGA) which proceeds in three subsequent steps: (i) a multilevel **γ**-clustering is used to identify the structure of the underlying network, (ii) the network is transformed to a tree, and (iii) finally, the resulting tree which shows the network structure is drawn using a variation of a force-directed algorithm. The algorithm has a potential to visualize very large networks because it uses modern clustering heuristics which are optimized for large graphs. Moreover, most of the edges are removed from the visual representation which allows keeping the overview over complex graphs with dense subgraphs.

## 1. Introduction

The development in systems biology has brought a strong interest in considering an organism as a large and complex network of interacting parts. Many subsystems of living organisms can be modeled as complex networks. One important example is a network of biochemical reactions which constitutes a complex system responsible for homeostasis in the living cell. An abstract network model of the biochemical processes within the cell can be constructed such that reactions are represented as nodes and metabolites (and enzymes) as edges. In the past, this system was studied mainly on a subsystem level through metabolic pathways. Recently, it has become important to consider the metabolic system as one complex network to understand deeper phenomena involving interactions across multiple pathways.

 The need to study the whole network consisting of thousands of reactions, metabolites, and enzymes requires a visualization system allowing biologists to study the overall structure of the system. Such a visualization should allow navigation and comprehension of the global system structures. In the present paper, we propose a visualization algorithm for very large networks arising in systems biology and we illustrate its usage on two complex biological networks. The first case study is a metabolic network of *Arabidopsis thaliana* and the second case study is a gene correlation network of *Mus musculus* based on mRNA expression measurements.

 Biological networks are usually represented as graphs because such model can provide an insight into their structure. The goal of the subsequent visualization is to present the information contained in the graph in a clear and structured way. For instance, closely related nodes of a subsystem should be positioned together. This can be achieved using a cost function which formalizes the visualization criteria and which controls the drawing algorithm. Several standard algorithms exist to achieve this goal using continuous optimization of the cost function, but the optimization of a discrete cost function remains hard to solve.

**Figure 1:**
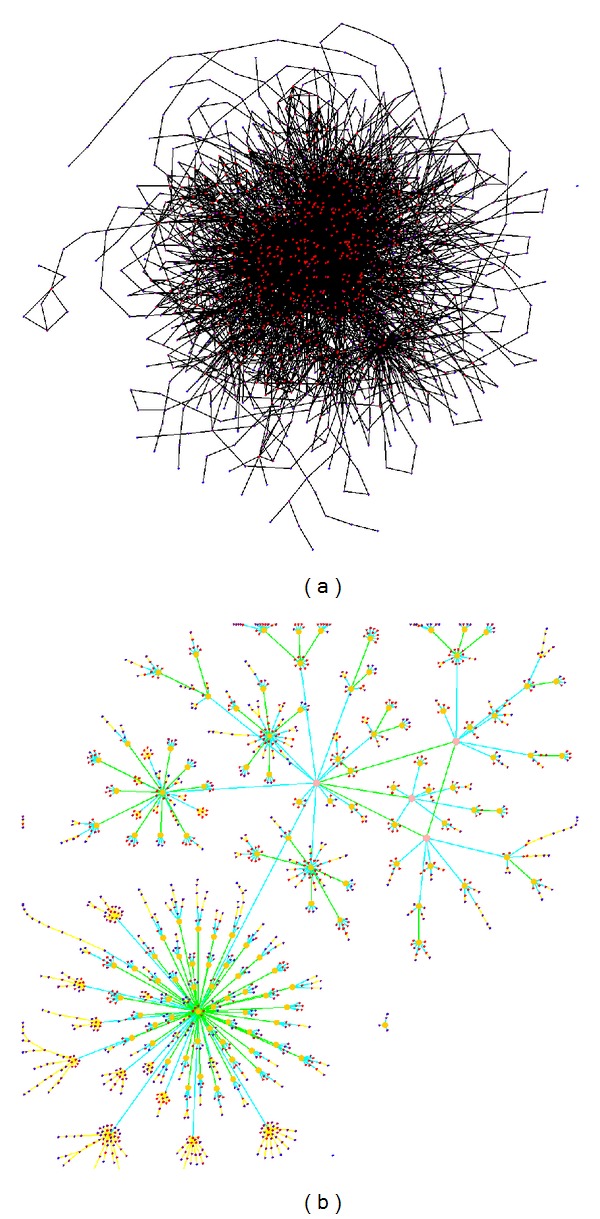
*Arabidopsis thaliana* metabolic network visualized with (a) a force-directed algorithm with all edges shown, (b) the MLGA method which combines *γ*-clustering with the force-directed algorithm. The underlying network has 1199 reactions (nodes) and 4386 metabolites (edges).

 A widely used graph drawing method for larger graphs is the force-directed layout algorithm [1, 2]. Basically, the graph is modeled as a physical system. A force is calculated on every node: a repulsive force between every pair of nodes and an attractive force if an edge exists between two nodes. The forces direct the system into a steady state which defines a final layout. However, the method has several disadvantages for large graphs with many edges. First, a straightforward implementation needs to calculate the forces between each node-pair in each iteration. Second, for complex graphs too many iterations are needed to find an optimal layout. Third, a drawback results from the node degree distribution in biological networks which tends to be skewed (scale-free). Few nodes have a high degree while a large number of nodes have a small degree. The attractive forces will stick together the nodes with many interactions in a small area which prevents the identification of the network structure in the dense parts of the network; see Figure 1(a). The repulsive force against the other nodes leads to a scattered layout. To overcome these disadvantages, other graph visualization methods have emerged which are discussed later. On the other hand, for a very specific class of graphs like trees, a modified version of force-directed algorithm can be still a suitable method.

 The visualization of very large biological networks was considered in [3]. The large graph layout algorithm (LGL) separates the graph into connected components, lays out each connected component separately, and integrates these layouts into one coordinate system. A grid variant of the spring-based algorithm [1] is used to draw the graph for each connected component. To separate dense parts of each component, the minimum spanning tree (MST) is calculated to define the order in which nodes are included in the layout computation. Beginning from a root node of the MST, new nodes with increasing edge distance from the root are iteratively added to the layout. The new nodes are placed randomly on spheres away from the current layout. At each iteration, the spring-based layout algorithm is executed until the layout is at rest. Under certain conditions, this node placement strategy reduces cluttering and retains the structure of core components; moreover, it separates highly connected components. This layout phase is illustrated in [3, page 181, Figure 1]. However, in some situations, the LGL algorithm can even obfuscate the true structure of the graph. Consider the situation where in the graph two cliques are connected by a matching. The MST algorithm will represent this subgraph as a star having many paths of length two from the center and one path of length three leading to the center of the second clique. The rendering according to LGL would lead to a situation where the first clique is placed in the interior of the second clique. Such starting configuration can easily lead to a situation where the force-directed algorithm cannot separate the cliques; moreover, the edges of the second clique would cross the rest of the graph. The problem is that in this situation the MST algorithm reduces the second clique to one edge. In such cases a different solution would be needed as we describe later.

 The problem of fast visualization for protein interaction networks was studied in [4]. The method uses an approach with a grouping phase, and a layout phase. In the grouping phase the algorithm identifies the connected components of the graph and uniformly selects pivot nodes in each component. The selection of the pivot nodes is controlled by a set of rules based on empirical parameters. In the layout phase, the pivot nodes define an initial layout of the connected components. Afterwards, the layout of each connected component is refined separately. The authors show that the method is faster than many other algorithms; however, a certain disadvantage of this algorithm is the choice of pivot nodes involving many parameters and a complex set of rules. The rules and its parameters are heuristically identified to give a uniform distribution of the nodes within the connected component. Another drawback is that the method per se cannot visualize, the structure of dense subgraphs because of too many edge crossings (see [4, page 1887, Figure 3]). To improve the visualization the authors introduce visual operations to collapse the cliques (and complete bipartite subgraphs) to reduce the number of edges and nodes. Additionally, the problem of finding maximal clique (or complete bipartite subgraph) is NP hard together with its approximation there is almost no chance to have fast identification heuristics for large graphs. Our algorithm improves the situation in this respect because relaxing requested density of the subgraph through *γ*-clustering (where 0 ≤ *γ* ≤ 1 is the cluster density) allows much more efficient heuristics for large graphs (order 10^6^ nodes and edges [5]).

 A global optimization method was explored in [6] where the authors describe a layout algorithm for metabolic networks. Nodes of the graph are placed on a square grid. A discrete cost function between a pair of nodes is introduced based on their relation and position on the grid. By minimizing the total cost, a layout is generated. A simulated annealing heuristic is used to optimize the cost function by choosing better layouts among possible candidates. Due to the computationally costly calculation of the layout, the approach is applicable to networks with a few hundred nodes only. The authors showed that the algorithm works well on sparse or planar graphs and clarifies the network structure as the cost function of the method places closely related nodes together. But this layout algorithm would place dense parts of the graph in the same area leading to many edge crossings. Additionally, as no reduction in the number of edges or nodes is performed, the identification of the graph structure would be very hard for large graphs with many edges.

## 2. MLGA Approach

 The experience with the existing visualization methods has shown that it is necessary to provide a structural view of dense networks. Representing networks with a large number of nodes and edges in a two-dimensional area results in many edge crossings. Dense subgraphs prevent the recognition of the network structure if drawn directly. Apart from other technical problems, this is the main shortcoming of most layout algorithms. We believe that the future progress in visualization of large and dense networks lies in algorithms which analyze the structure of the graph first and then generate a new graph which contains specific semantic symbols for regular substructures like dense clusters. Additionally, the algorithms may allow for drilling down and interactively show all edges for a given substructure, described below (see section visual representation and operation). Dense clusters are ideal candidates for graph preprocessing because they can be simply described, efficiently searched, and if they are replaced with a specific symbol they significantly reduce the complexity of the resulting low-dimensional (planar or three dimensional) picture because they contain most of the edges. Moreover, we focus on the graph clustering algorithms because the underlying dimension of graphs can be very high providing difficulties for other clustering algorithms.

 Graph clustering is a large field with many algorithms developed over the years [7]; however, there is no universal solution for all cases. Even a definition of a cluster comes in many flavors with different algorithmic consequences. Therefore, it is important to consider a certain class of graphs which is sufficiently general in the context of bioinformatics but allows for using an efficient clustering method. Recently new clustering methods emerged based on the idea of so-called *γ*-clusters [5] or (*α*, *β*)-clusters [8]. These methods use fast heuristics which allow for clustering efficiently large graphs. The existence of such methods inspired the general idea behind our research to use clustering algorithms to build a hierarchical structure of a given graph which can be much better visualized and which tells the users more about the structure of the underlying biological network. In the following, we focus on *γ*-clusters but other graph clustering methods could be used as well.

## 3. Algorithm

**Figure 2:**
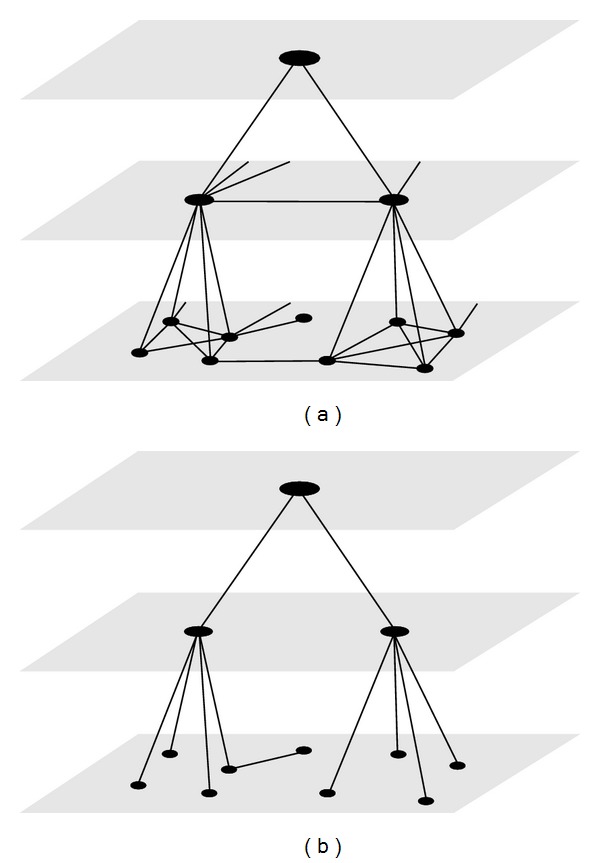
(a) The construction of a cluster hierarchy and (b) the transformation to a tree.

 The MLGA method introduces multilevel *γ*-clustering and a specific tree transformation with a force-directed layout algorithm to visualize the structure of highly complex biological networks. First, the original graph is preprocessed using a *γ*-clustering algorithm described in [5] to identify the clusters. For every cluster, a new cluster node is created and these new nodes are linked with new edges if there are edges between the underlying cluster nodes as illustrated in Figure 2.

This process constructs the first hierarchical layer above the original graph. Then, the clustering algorithm is recursively applied to the cluster nodes itself to generate a cluster hierarchy. Afterwards, this hierarchy is transformed to a tree showing only the shortest paths from a root node through the intermediate cluster nodes to the nodes of the initial graph. Finally, a modified version of the force-directed algorithm visualizes the tree structure of the remaining graph. This combination of preprocessing and layout algorithm eases the identification of the cluster structure and their interactions, see Figure 1(b).

For the clustering step, we prefer *γ*-clustering to (*α*, *β*)- clustering or to other more complex methods because it would be much more difficult to control the clustering parameters during the transitions between the hierarchy levels. The only parameter which has to be specified for our algorithm at every hierarchical level is the parameter *γ*. It can be seen that the density of the graph grows when the algorithm proceeds to the higher levels. On the other hand, the number of nodes decreases very rapidly so that after few steps there is only one clique left. As a consequence, it is not meaningful to use the same clustering parameters as the algorithm recursively proceeds up the hierarchy. For more complex clustering algorithms, it would be very difficult to define a good clustering parameters if the parameter space has more dimensions. In our case, the sequence of the values for the parameter *γ* must be growing. As we discuss later, the actual values can be empirically determined and moreover 3-4 values are sufficient for large graphs.

## 4. Algorithmic Phases

Let *G* = (*V*, *E*) be an undirected graph *G* with the vertex set *V* and edge set *E*. A *γ*-cluster for 0 ≤ *γ* ≤ 1, also described as *γ*-clique or dense subgraph, is a subset *S*⊆*V* such that for its edge set *E*(*S*) and the vertex set *V*(*S*) the following is true:

(1) $$\begin{array}{c} \left|E\left(S\right)\right|\geq \gamma \left(\begin{array}{c} \left|V\left(S\right)\right|\\ 2 \end{array}\right). \end{array}$$

Finding a *γ*-clique of maximal cardinality in *G* is the maximal *γ*-clique problem. The 1-clique problem is NP hard and is proved to be hard to approximate [9].

To identify the clusters on one hierarchical level, we use a heuristic developed in [5] to detect *γ*-clusters for very large graphs. Reference [5] introduced a potential function on a vertex set relative to a given *γ*-cluster and derived an algorithm to discover maximal *γ*-clusters. The time complexity of the algorithm is *O*(|*S*||*V*|^2^) with *S* the set of vertexes of the maximal *γ*-cluster detected. Further, the authors use a greedy randomized adaptive search procedure (GRASP) version of the algorithm with edge pruning. The feasibility of the resulting method was demonstrated by applying it to telecommunication data with millions of vertexes and edges.

## 5. *γ*-Cluster Detection

To find all *γ*-clusters on one level in the graph a variant (Algorithm 1) of the GRASP approach of [5] is used. The cluster construction procedure *construct*_*dsubg* is the nonbipartite case for finding a high cardinality cluster of specified density *γ* in a graph with nodes *V* and edges *E*. Our algorithm repeatedly applies the detection algorithm to the highest hierarchical level of the new graph. It terminates if no more *γ*-clusters are found or the number of clusters with a cluster-size below a given minimum size is reached.

## 6. Hierarchy Creation

The cluster detection algorithm is repeatedly applied to the graph and the clusters to build a hierarchy; see Figure 2(a). Each node of the graph has an attribute level which is initially assigned to zero. First, the cluster detection algorithm retrieves the clusters of this initial graph. Afterwards, the algorithm iteratively creates the clusters of the next level *i* among the clusters one level below *i* − 1 (Algorithm 2). To control the density of the clusters on each level a *γ*_*i*_-value is specified. At each level an edge between the cluster nodes is created if an edge exists between the nodes one level below. Additionally, a new edge is generated between the cluster and the nodes belonging to the cluster. The algorithm terminates if no more clusters are found. This phase resembles hierarchical clustering where new nodes are introduced for hierarchically different clusters but the *γ*-clustering is based on a completely different density measure and merges multiple nodes in one step. Consequently, this leads to a much lower tree depth (as described in the following section) compared to the hierarchical clustering which generates a binary tree.

## 7. Tree Transformation

To gain the structure of the cluster hierarchy, a tree transformation is performed; see Figure 2(b). In the transformation (Algorithm 3), a hidden root node is connected to all cluster nodes at the highest level as their parent. Afterwards, only the edges belonging to the shortest path from the root node to each node is shown. If the shortest path is not unique a path will be chosen at random. The distance for each node is calculated beginning from the root using a breath-first search. The parent of a node will be set to the neighbor node with the shortest distance. If the node belongs to a cluster node at one level above, the parent is set to this cluster.

## 8. Layout Algorithm

A modified version of a force-directed algorithm [2] is used to lay out the transformed graph. Our method introduces different edge length on each level. Longer edges are assigned to higher levels than on lower levels. This results in a natural visualization of the hierarchy. Furthermore, the initial positions of the nodes are specifically calculated. The nodes of the graph are located on concentric circles with the hidden root node at the center. Nodes immediately connected to the root are positioned at the next inner circle and so on. A segment of the circle is assigned to each node within which its location is calculated. Recursively, a fraction of this segment is assigned to the children of the node on the next circle. This initial setup reduces the rendering time and guides the layout algorithm to visualize the tree structure. A random initial positioning may result in a local minimum of the force-directed layout with many edge crossings which would disrupt the tree representation. Additionally, the repulsive forces are ignored beyond a given distance depending on the size of the drawing area. This restriction prevents disconnected components of the graph from separating too far. To suppress the well-known oscillation problem [10] of force-directed algorithms a dumping heuristics is used where we compute an average of the previous node positions during the force calculation.

As the graph is transformed to a tree, other layout algorithms can be used in the this phase. Reference [11] uses a level-based approach which horizontally aligns nodes with the same distance from the root node. As only a few levels are created for our initial graph the resulting drawing would have a much larger width than height. A ringed circular approach like [12] where the children of the nodes are plotted on the periphery of a circle has a better space efficiency on the 2D plane than [11]. But a visual inspection of the resulting graph in [12, page 11, Figure 7 (left)] shows that the force-directed layout distributes the children of a node more evenly.

## 9. Visual Representation and Operation

**Figure 3:**
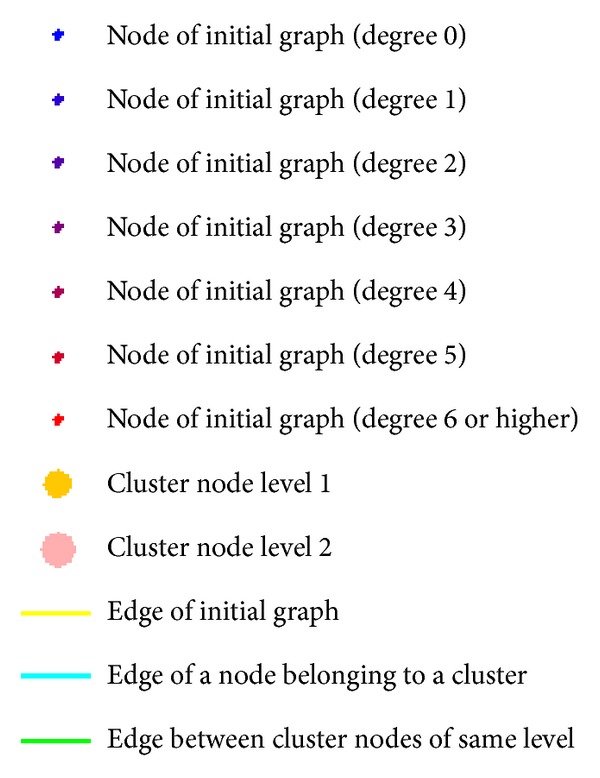
Semantic symbols used in MLGA visualization. Nodes and edges are color encoded, nodes of the initial graph are colored according to their degree and cluster nodes are enlarged at each level of the hierarchy.

After the creation of the cluster hierarchy and the tree transformation many initial edge connections are hidden. In the presentation of the resulting graph the nodes of the inital graph are colored depending on the number of edges in the initial graph. Additionally cluster nodes and edges are visualized with different symbols and colors (Figure 3). 

**Figure 4:**
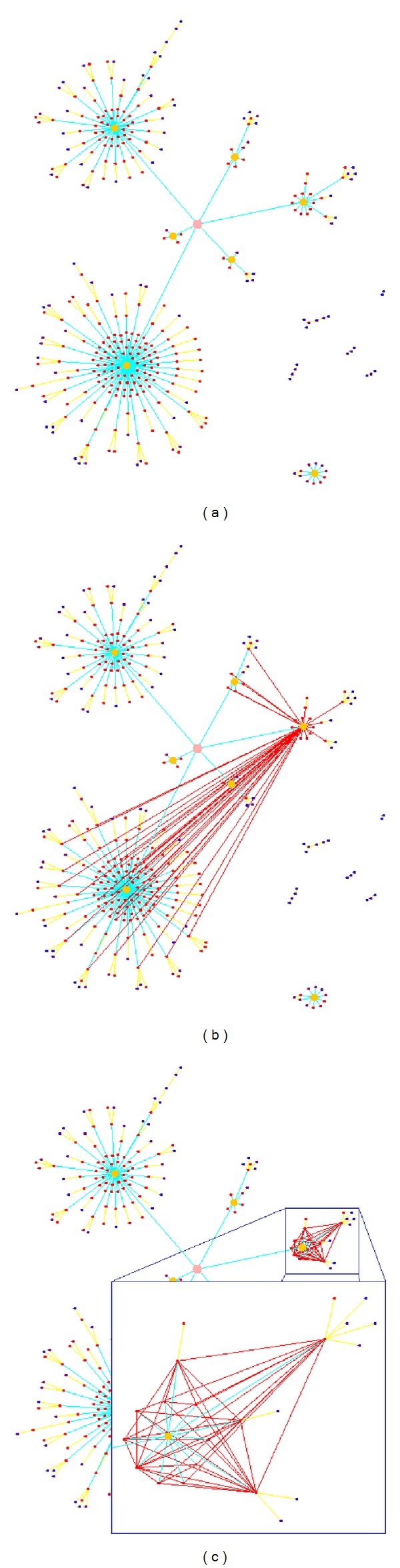
(a) A part of a gene correlation network of *Arabidopsis thaliana* drawn with MLGA, (b) showing all edges connected to the *γ*-cluster node at the top right and (c) displaying all edges between the nodes defining the cluster. The inset shows a magnification of the edges of the selected cluster.

Our implementation of the visualization tool offers two operations to get deeper insight into the original graph. First, all edges between a selected node and its direct neighbors can be highlighted (Figure 4(b)). If the marked node is a cluster node, all connections to the nodes of the cluster will be shown. During the tree transformation, most of these connections were eliminated and a direct connection between two nodes in different clusters was replaced by an indirect connection between the cluster nodes. The second operation will display all edges between the nodes forming a *γ*-cluster node (Figure 4(c)) which allows the user to temporarily alter the view between the star-shaped cluster node and the real connections of the cluster.

## 10. Computation Speed and Memory Requirements

Retrieving a *γ*-clique with the clustering algorithm of [5] has a running time of *O*(|*S*||*V*|^2^) with |*S*| the size of the detected clique and |*V*| the size of the initial graph. As the algorithm is recursively applied to the remaining nodes of the graph, a time complexity of at most *O*(|*V*|^3^) results on each level of the hierarchy. The number of levels depends on the number of clusters found on each level. It ranges from the worst case where two nodes are clustered together log⁡_2_|*V*| down to 1 if all nodes are in the same cluster. Therefore, the total runtime order has an upper limit *O*((log⁡_2_|*V*|)^4^) and a lower limit *O*(|*V*|^3^)  . The tree transformation of the resulting hierarchy uses breath-first search. As new nodes and edges are introduced during the hierarchy creation its runtime ranges from *O*(4|*V*| + |*E*|) down to *O*(|*V*| + |*E*|) in terms of the inital number of nodes |*V*| and initial number of edges |*E*|.

The implemented version of force-directed layout algorithm needs a runtime of *O*(|*V*|^2^). A specialized tree layout algorithm like [11] has a runtime of order *O*(|*V*|) and [12] an order *O*(|*V*|) or *O*(|*V*|log⁡|*V*|) if an optimal solution for the circle size is required.

The memory required by the algorithm mainly depends on the graph representation. For biological networks a representation between the worst case *O*(4|*V*| + |*E*|) and *O*(|*V*| + |*E*|) space is suitable.

## 11. Results

### 11.1. Metabolic Networks

**Figure 5:**
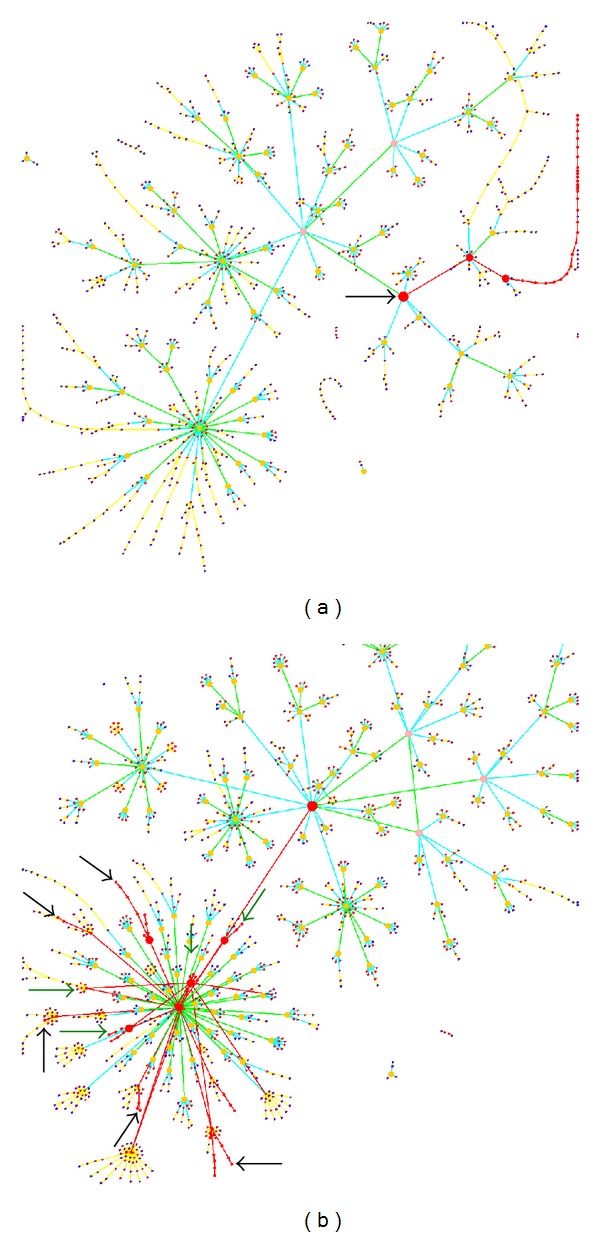
MLGA applied to (a) A. thaliana biochemical network without signaling effects and regulatory elements. Reactions directly involved in the synthesis of brassinosteroids are highlighted with the red color and direct connections are depicted by red edges. The level 2 cluster, indicated by an arrow, combines the major parts of isoprenoid biosynthesis, resulting from the nonmevalonate pathway. (b) A. thaliana biochemical network including signaling effects and regulatory elements. Reactions directly involved in brassinosteroid and auxin metabolism/signaling are highlighted with red and direct connections are depicted by red edges. Black arrows point to reactions involved in brassinosteroid metabolism/signaling. Green arrows point to reactions involved in auxin metabolism/signaling.

 To provide experimental justification of the proposed method, we extracted the metabolic network for *Arabidopsis thaliana* from Genevestigator [13]. The network has 932 nodes and 2315 edges. The edges represent metabolites and the nodes represent biochemical reactions. We used two versions of the network as illustrated in Figure 5(a) where the second version in Figure 5(b) contains additionally the regulatory pathways with enzymes as edges leading to 1199 nodes and 4386 edges.

 The application of multi-level *γ*-clustering to visualize the *A. thaliana* biochemical network (Figure 5(a)) revealed that both global view and lucidity were sustained, which is also true when regulatory pathways were added to the network (Figure 5(b)). We looked at plant isoprenoid biosynthesis, in particular at the synthesis of brassinosteroids (BRs), a class of plant hormones which are essential for the regulation of plant growth and development [14, 15]. All individual reaction steps, leading to BRs, were found structured according to metabolite flux through the pathway as part of a level 2 cluster, also containing upstream pathways as well as reactions leading to other isoprenoid end products (Figure 5(a)). After inclusion of regulatory elements and signal-transduction chains, multi-level *γ*-clustering assigned the biochemical reactions from brassinosteroid biosynthesis to clusters containing reactions known to be regulated by BRs and elements that are involved in the regulation of this biosynthetic pathway. As an example, the known fact that BRs act synergistically with auxins to promote cell elongation [16] is nicely reflected in the MLGA drawn network (Figure 5(b)).

### 11.2. Gene Interaction Networks

The MLGA method can also be successfully used to analyze gene interaction networks. Gene interaction networks are constructed as graphs where nodes represent genes and edges represent interactions between genes on various biological levels, as for example, interactions between the corresponding proteins or regulatory and causal interactions obtained from gene expression experiments. There is a long-term research into methods how to obtain networks which identify different kinds of gene interaction networks based on different types of input data [17–22]. However, as we illustrate later, even if a simplified network generation method was used, our visualization algorithm was able to identify correctly the biologically meaningful subsystems from a genomewide correlation network.

**Figure 6:**
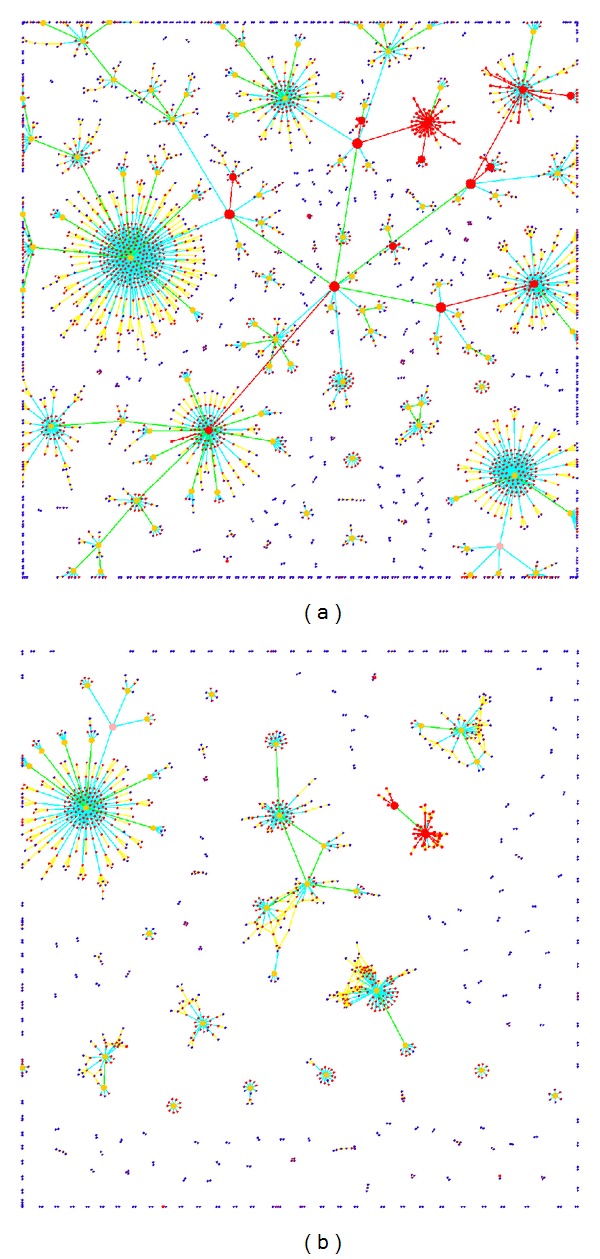
MLGA applied to (a) *Mus musculus* gene correlation network generated with a threshold of 0.72. (b) The gene correlation network generated with a threshold of 0.80. The red highlighted nodes and direct connections belong to the ribosomal cluster.

 To generate the gene correlation networks, we used a *Mus musculus* dataset from the Genevestigator database. The data consisted of 3157 publicly available Affymetrix arrays. Each array measured the expression values of 12488 genes. The gene correlation matrix was calculated using the Pearson correlation and afterwards a network was generated, where an edge was introduced between two genes if the correlation value between the genes was above a certain threshold. Two networks were constructed using a threshold of 0.72 in Figure 6(a) and 0.80 in Figure 6(b).

 We use a well-known ribosomal gene complex [23–25] to illustrate the possibilities of MLGA to discover interesting structures in the correlation network. An inspection of the cluster highlighted in Figure 6(a) shows that it contains genes which are documented to belong to the ribosomal cluster. Moreover, our method has a structural stability in a wide range of graph density. This can be seen comparing Figures 6(a) and 6(b). Figure 6(a) has lower threshold; therefore, it contains 38 889 edges and 2774 nodes compared to 8659 edges and 1232 nodes in Figure 6(b). In both cases the ribosomal cluster can be clearly identified.

## 12. Discussion

 Visualization methods often contain parameters which must be empirically identified. In [4], the selection of pivot nodes is determined by a set of empirical values to achieve a uniform distribution of these nodes in the network. Afterwards, the layout is computed with respect to the selected pivot nodes. In our method, the only important empirically set parameters are the cluster densities *γ* on each hierarchical level. This influences the granularity of the visualization and the subsequent tree transformation supports the recovering of the network structure. The computational experience has shown that it is recommended to use a slightly smaller *γ* value in the first than in the subsequent levels; we use 0.5 and 0.7, respectively. The density of the graph grows considerably over the hierarchical levels. In most of the experiments with a graph sizing up to 10^5^ edges the third level was already a clique. Therefore, very few *γ* values are needed even for large graphs.

 A different approach in a similar direction as our research is described in [26]. The authors consider weighted graphs where vertexes with degree 1 are repeatedly removed until no more vertex of degree 1 exists. The removed nodes can be added at the end of the drawing algorithm. After that, a hierarchy of clusters is calculated on the remaining graph using an approximation of the graph distances. A cluster is formed if the pairwise shortest path of its nodes is equal to or above a threshold which depends on the hierarchy level. This leads to different definition of clusters than in our algorithm together with a different cluster heuristics. The algorithm preserves more edges than in our case making the structure of the graph more difficult to identify. Comparing the result of MLGA Figure 1(b) with [26], the structure of graphs in [26] is only partially resolved (see, e.g., [26, page 2, Figure 1]). Similarly as in our case a version of force-directed algorithm is used in the last stage to refine the visualization. A higher weight is assigned to the edges between the nodes of a cluster than to the edges between clusters. This forces the nodes of a cluster to be drawn close to each other. In our approach, the additional cluster node and the desired edge length have a similar effect.

 In addition to complexity problems for large graphs (NP-hard approximation), the algorithms based on identification of cliques have also a drawback that it is often not clear which cliques are relevant. This problem is particularly present in cases of graphs with dense subgraphs, where we obtain a system of large cliques with similar sizes which have additionally large intersections. In particular, in the presence of noise where every measurement defines a graph which differs in a small percentage of edges, it is difficult to decide during visualization based on cliques which part of the graph can be emphasized as structurally important. An interesting development is represented by [27] where the authors concentrate on intersections of large clusters under a condition that these intersections are cliques. They identify so-called “atom subgraphs” which represent clique minimal separator decomposition (a separator is a set of vertexes whose removal will disconnect the graph into several parts). However, the relaxation from cliques to dense clusters in our method improves also on the intersection problem because our method would glue the cliques with large intersection into one cluster.

## 13. Conclusion

 As discussed, many approaches try to improve the layout of complex networks through better placement of the nodes alone. In our work, we pursue a different line of research towards efficient visualization algorithms for large biological networks. Our approach does not aim at rendering all edges in a network, but we focus on the discovery and visualization of important structural features. This approach is combined with complementary visual operations which allow to drill-down into the details of structurally identified elements. The MLGA method is successful in identifying the biologically relevant structures and allows for processing very large graphs as we illustrated on two different case studies of biological networks. Naturally, this paradigm opens new questions on how to further improve the visualization output and speed. Different clustering algorithms can be tried to create the multi-level structure; however, in the case of multiparameter clustering the control and analysis of the parameter values between the levels would become more difficult.

 On the theoretical side, the next question to consider is how to provide a provably good (optimal) sequence of *γ* values. Another question is whether the surprisingly good structure identification features of our algorithm could be traced back to the scale-free character of many biological networks.

## Figures and Tables

**Algorithm 1:**
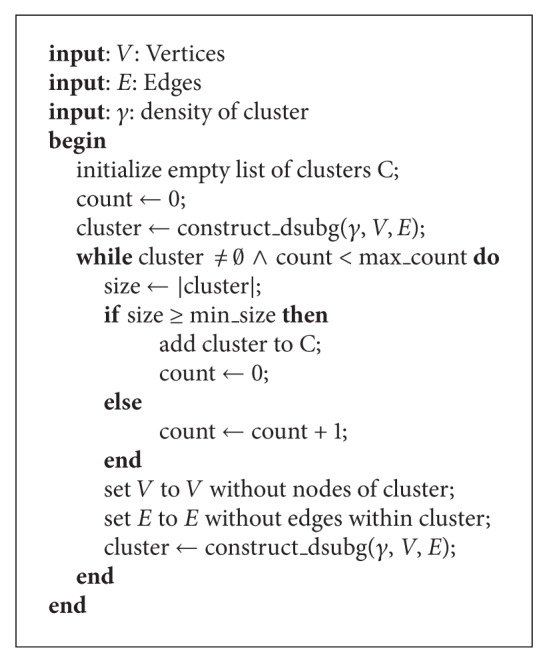
createClusters.

**Algorithm 2:**
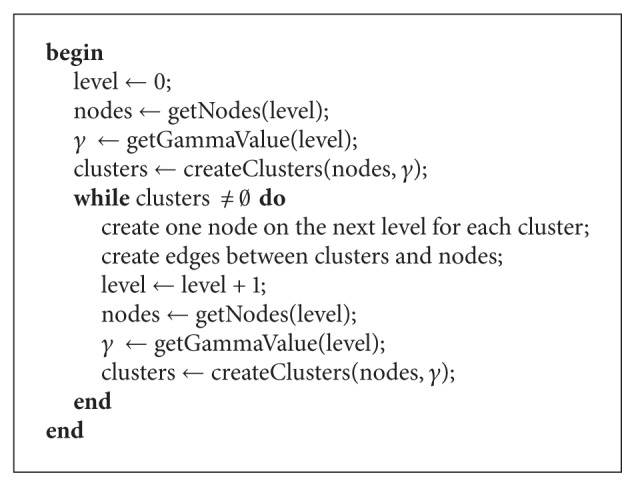
createMultiLevelClusters.

**Algorithm 3:**
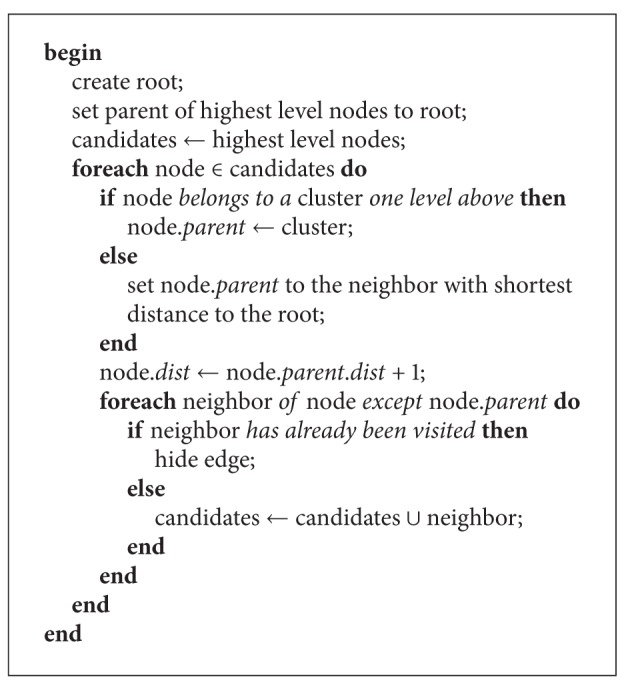
treeTransformation.

## References

[1] Kamada T, Kawai S (1989). An algorithm for drawing general undirected graphs. *Information Processing Letters*.

[2] Ffuchterman TMJ, Reingold EM (1991). Graph drawing by force-directed place-ment. *Software*.

[3] Adai AT, Date SV, Wieland S, Marcotte EM (2004). LGL: creating a map of protein function with an algorithm for visualizing very large biological networks. *Journal of Molecular Biology*.

[4] Han K, Ju B-H (2003). A fast layout algorithm for protein interaction networks. *Bioinformatics*.

[5] Abello J, Resende M, Sudarsky S, Rajsbaum S (2002). Massive quasi-clique detection. *LATIN 2002: Theoretical Informatics*.

[6] Li W, Kurata H (2005). A grid layout algorithm for automatic drawing of biochemical networks. *Bioinformatics*.

[7] Schaeffer SE (2007). Graph clustering. *Computer Science Review*.

[8] Mishra N, Schreiber R, Stanton I, Tarjan R, Bonato A, Chung F (2007). Clustering social networks. *Algorithms and Models For the Web-Graph*.

[9] Hastad J (1999). Clique is hard to approximate within n^1-*ε*^. *Acta Mathematica*.

[10] Frick A, Ludwig A, Mehldau H, Tamassia R, Tollis I (1995). A fast adaptive layout algorithm for undirected graphs (extended abstract and system demonstration). *Graph Drawing*.

[11] Reingold EM, Tilford JS (1981). Tidier drawings of trees. *IEEE Transactions on Software Engineering*.

[12] Grivet S, Auber D, Domenger JP, Melancon G, Wojciechowski K, Smolka B, Palus H, Kozera R, Skarbek W, Noakes L (2006). Bubble tree drawing algorithm. *Computer Vision and Graphics*.

[13] Hruz T, Laule O, Szabo G (2008). Genevestigator v3: a reference expression database for the meta-analysis of transcriptomes. *Advances in Bioinformatics*.

[14] Asami T, Min YK, Sekimata K (2001). Mode of action of brassinazole: a specific inhibitor of brassinosteroid biosynthesis. *ACS Symposium Series*.

[15] He J-X, Gendron JM, Yang Y, Li J, Wang Z-Y (2002). The GSK3-like kinase BIN2 phosphorylates and destabilizes BZR1, a positive regulator of the brassinosteroid signaling pathway in Arabidopsis. *Proceedings of the National Academy of Sciences of the United States of America*.

[16] Halliday KJ (2004). Plant hormones: the interplay of brassinosteroids and auxin. *Current Biology*.

[17] Yip KY, Alexander RP, Yan K-K, Gerstein M (2010). Improved reconstruction of in silico gene regulatory networks by integrating knockout and perturbation data. *PLoS ONE*.

[18] Mutwil M, Usadel B, Schütte M, Loraine A, Ebenhöh O, Persson S (2010). Assembly of an interactive correlation network for the Arabidopsis genome using a novel Heuristic Clustering Algorithm. *Plant Physiology*.

[19] Marbach D, Prill RJ, Schaffter T, Mattiussi C, Floreano D, Stolovitzky G (2010). Revealing strengths and weaknesses of methods for gene network inference. *Proceedings of the National Academy of Sciences of the United States of America*.

[20] De Bodt S, Proost S, Vandepoele K, Rouzé P, Van de Peer Y (2009). Predicting protein-protein interactions in Arabidopsis thaliana through integration of orthology, gene ontology and co-expression. *BMC genomics*.

[21] Rhodes DR, Tomlins SA, Varambally S (2005). Probabilistic model of the human protein-protein interaction network. *Nature Biotechnology*.

[22] de la Fuente A, Bing N, Hoeschele I, Mendes P (2004). Discovery of meaningful associations in genomic data using partial correlation coefficients. *Bioinformatics*.

[23] Rual J-F, Venkatesan K, Hao T (2005). Towards a proteome-scale map of the human protein-protein interaction network. *Nature*.

[24] Ishii K, Washio T, Uechi T, Yoshihama M, Kenmochi N, Tomita M (2006). Characteristics and clustering of human ribosomal protein genes. *BMC Genomics*.

[25] Atias O, Chor B, Chamovitz DA (2009). Large-scale analysis of Arabidopsis transcription reveals a basal co-regulation network. *BMC Systems Biology*.

[26] Bourqui R, Auber D, Mary P How to draw clustered weighted graphs using a multilevel force-directed graph drawing algorithm.

[27] Kaba B, Pinet N, Lelandais G, Sigayret A, Berry A (2007). Clustering gene expression data using graph separators. *In Silico Biology*.

